# Are summer schools a way to improve recruitment in psychiatry?

**DOI:** 10.1192/bjb.2020.77

**Published:** 2021-04

**Authors:** Aileen O'Brien

**Affiliations:** 1 St. George's University of London, UK

**Keywords:** Education and training, stigma and discrimination, recruitment, ChoosePsychiatry, widening participation

## Abstract

Summer schools are traditionally used to encourage sixth form students to consider a career in medicine. Is it worth attracting students earlier in their school career, concentrating on psychiatry? Wyke et al describe an innovative project attempting to do just that.

Summer schools – where prospective students are invited to a university or other setting during the holidays – are a well-established way of encouraging students to consider applying for a particular course. They are usually a week long and are aimed at particular groups of students, for example, international students or those from more deprived backgrounds. Although they are traditionally held on campus, there would be scope in the future to run them remotely, depending on the COVID-19 situation.

Medicine has traditionally been and remains a competitive course, but medical schools are also in competition with each other to attract students. The most prized students for financial reasons are international students, as they pay higher fees (one of the reasons COVID-19 presents a financial risk to many universities).

The widening participation agenda – attracting students from more deprived backgrounds – is another financial inducement for universities. Unless universities can prove their commitment to this, which is surprisingly hard to measure and evidence, they are not allowed to charge high-rate tuition fees. Students from such backgrounds are a group often targeted for invitation to summer schools by universities.

The #ChoosePsychiatry campaign has tried to encourage doctors to choose the specialty – and to an admirable extent has succeeded, with rates of juniors going into psychiatry increasing.^1^ The campaign to choose psychiatry includes a target audience of sixth formers who have already chosen to study medicine. As the conversation about mental health, especially post COVID-19, becomes part of the national *zeitgeist*, are we missing a trick in not trying to interest psychologically minded students into medicine earlier than sixth form? Wyke et al describe an innovative 1 week summer school for GCSE students, not all of whom had decided on medicine as a potential career.^2^ The week included talks from psychiatrists at different levels of training, groups and debates, and the students met patients and medical students. At the end of the week, students were more likely than before to choose psychiatry as a career, had changed their views regarding social restriction in mental health and had uniformly positive attitudes towards the course.

Some of these students will presumably have gone to the summer school in order to build their CV, having already decided to apply for medicine, but who knows whether a psychiatry spark has been lit in a budding doctor who wouldn't have considered the specialty otherwise?

It may be well be that the resources and expense required for the project, which were not evaluated in Wykes's paper, are not worth the long-term results. It will be very interesting to see how many of these teenagers do study medicine and choose psychiatry. We know that many students interested in psychiatry at the start of medical school are put off by the ‘badmouthing’ of the specialty by their educators and peers,^3^ so hopefully those enthusiastic students will not have their initial enthusiasm knocked out of them along the way. Others may ultimately decide not to study medicine, or to study medicine but not choose psychiatry; if so, at least a group of bright adolescents have had their eyes opened to the subject and had stigmatising clichés about psychiatry challenged.

## About the author

**Aileen O'Brien** is a Reader in Psychiatry and Education at St. George's University of London, UK.

## References

[1] Royal College of Psychiatrists. *How to Become a Psychiatrist*. RCPsych (https://www.rcpsych.ac.uk/become-a-psychiatrist/choose-psychiatry/how-to-become-a-psychiatrist).

[2] Wyke C, de Bernier GL, Sin Fai Lam C, Holt C, Butler S, Rajkumar AP, Perspectives of GCSE students attending a psychiatry summer school in south London. BJPsych Bull 2020; this issue. 10.1192/bjb.2020.76 PMC8112016 33762046

[3] Ajaz A, David R, Brown D, Smuk M, Korszun A. BASH: badmouthing, attitudes and stigmatisation in healthcare as experienced as medical students. BJPsych Bull 2016; 40(2): 97–102. 27087996 10.1192/pb.bp.115.053140 PMC4817656

